# Increased Muscle Activity Accompanying With Decreased Complexity as Spasticity Appears: High-Density EMG-Based Case Studies on Stroke Patients

**DOI:** 10.3389/fbioe.2020.589321

**Published:** 2020-11-16

**Authors:** Tian Xie, Yan Leng, Yihua Zhi, Chao Jiang, Na Tian, Zichong Luo, Hairong Yu, Rong Song

**Affiliations:** ^1^Key Laboratory of Sensing Technology and Biomedical Instrument of Guangdong Province, School of Biomedical Engineering, Sun Yat-sen University, Guangzhou, China; ^2^Department of Rehabilitation Medicine, Guangdong Engineering Technology Research Center for Rehabilitation Medicine and Clinical Translation, The First Affiliated Hospital, Sun Yat-sen University, Guangzhou, China; ^3^Department of Electromechanical Engineering, Faculty of Science and Technology, University of Macau, Macau, China

**Keywords:** spasticity, entropy, HD-EMG, stroke, stretch reflex

## Abstract

Spasticity is a major contributor to pain, disabilities and many secondary complications after stroke. Investigating the effect of spasticity on neuromuscular function in stroke patients may facilitate the development of its clinical treatment, while the underlying mechanism of spasticity still remains unclear. The aim of this study is to explore the difference in the neuromuscular response to passive stretch between healthy subjects and stroke patients with spasticity. Five healthy subjects and three stroke patients with spastic elbow flexor were recruited to complete the passive stretch at four angular velocities (10°/s, 60°/s, 120°/s, and 180°/s) performed by an isokinetic dynamometer. Meanwhile, the 64-channel electromyography (EMG) signals from biceps brachii muscle were recorded. The root mean square (RMS) and fuzzy entropy (FuzzyEn) of EMG recordings of each channel were calculated, and the relationship between the average value of RMS and FuzzyEn over 64-channel was examined. The two groups showed similar performance from results that RMS increased and FuzzyEn decreased with the increment of stretch velocity, and the RMS was negatively correlated with FuzzyEn. The difference is that stroke patients showed higher RMS and lower FuzzyEn during quick stretch than the healthy group. Furthermore, compared with the healthy group, distinct variations of spatial distribution within the spastic muscle were found in the EMG activity of stroke patients. These results suggested that a large number of motor units were recruited synchronously in the presence of spasticity, and this recruitment pattern was non-uniform in the whole muscle. Using a combination of RMS and FuzzyEn calculated from high-density EMG (HD-EMG) recordings can provide an innovative insight into the physiological mechanism underlying spasticity, and FuzzyEn could potentially be used as a new indicator for spasticity, which would be beneficial to clinical intervention and further research on spasticity.

## Introduction

Spasticity, one of the positive features of the upper motor neuron syndrome (UMNS) after stroke or other central nervous system diseases, is a velocity-dependent increase in muscle tone caused by an exaggeration of stretch reflex (Lance, 1980). The prevalence of spasticity ranges from 25 to 40% within the year after the first-ever stroke (Watkins et al., 2002; Sommerfeld et al., 2004; Urban et al., 2010; Wissel et al., 2010). Spasticity contributes significantly to motor impairments and activity limitations for stroke patients. Not only does it cause pain and many secondary complications, such as urinary incontinence, pressure ulcers and contractures, but it can also interfere with walking, sitting and voluntary movements of limbs, seriously affecting the quality of life (QoL) of patients (Hsu et al., 2003; Duncan et al., 2005; Welmer et al., 2006; Francisco and McGuire, 2012; Ward, 2012). Effective and targeted intervention for spasticity is critical for neurorehabilitation after stroke. A clear understanding of the pathophysiology of spasticity may facilitate the development of its clinical management.

An accurate and objective assessment tool is a reliable guarantee for investigating the underlying mechanism of spasticity. In order to make up for the subjectivity and poor reliability of commonly used clinical scales, such as the Ashworth Scale (AS) and Modified Ashworth Scale (MAS), some studies used the isokinetic dynamometer to assess the spasticity by quantifying the resistance of a joint to passive stretch (Kim et al., 2005; Starsky et al., 2005; Damiano et al., 2007). Matching the velocity-dependent characteristics, this biomechanical measure can standardize the subject’s range of motion (ROM) to carry out passive stretch at a predefined constant angular velocity. Compared with the use of clinical scales, using this method is not only objective and accurate, but also has better reliability and validity (Pierce et al., 2006; Sin et al., 2019). However, the resistance is composed of two parts, namely the neural component of the tonic stretch reflex and the non-neural component of the soft tissue characteristics (Fleuren et al., 2010; Li et al., 2017). After UMN lesions, the non-neural component also changes and depends on the stretch velocity (Lindberg et al., 2011). Hence this quantification is difficult to specifically distinguish the contribution of the velocity-response of stretch reflex to the increased resistance. On the other hand, the electrophysiological measure, which is not affected by mechanical components, has superiority in representing the velocity-dependent reflex components to assess spasticity (Kim et al., 2005; Zhang et al., 2019). On the basis of electromyography (EMG), the reflex muscle activity in response to passive stretch can be well reflected (Kim et al., 2005; Voerman et al., 2005; Wood et al., 2005; Sorinola et al., 2009). Recently, the innovation of high-density EMG (HD-EMG) recording the EMG signals in a wide area with dense electrodes of 2D arrays, has the potential to provide spatial features in addition to temporal features of muscle activity to study the neuromuscular function (Rojas-Martínez et al., 2012). The spatial distribution can describe the activation of different portions within regions of a muscle, which increases the possibility of capturing the motor units’ (MUs) characteristics (Drost et al., 2006; Gallina and Botter, 2013). The additional ability enhances the discrimination power of differences of action potentials between two MUs, which is more sensitive than intramuscular electrodes. As a result, it is feasible to extract individual MU property from surface EMG (Zhou et al., 2011). By constructing the activation map obtained from 2D arrays recordings, the HD-EMG technique is able to track the spatial heterogeneity of muscle activity during different tasks (Castroflorio et al., 2012; Hu et al., 2014, 2015). The heterogeneous activation underlines the inhomogeneous distribution and recruitment of MUs in the muscle area (Farina et al., 2008). A recent study revealed that after injection of botulinum toxin, the muscle activity of a stroke patient with spasticity was not homogeneous during voluntary elbow flexion tasks (Afsharipour et al., 2018), indicating possible changes in spatial recruitment patterns within the spastic muscle. However, it is unclear whether the activation pattern of involuntary muscle activity changes in the generation of spasticity. The spatial distribution can identify changes in muscle activity during passive stretch under the influence of spasticity, which provides a unique perspective for understanding its underlying mechanism. Applying the combination of isokinetic dynamometer and HD-EMG in the measurement, we can not only reliably assess the muscular reflex response to passive stretch, but also explore possible alterations in the spatial distribution of reflex activation due to spasticity.

In addition, considering that the EMG signals are complex, different types of entropy indexes have been developed to capture its non-linear features. Compared with the approximate entropy (ApEn) and sample entropy (SampEn), fuzzy entropy (FuzzyEn) has advantages in robustness and data length dependence, which could characterize the regularity of the neuromuscular system more reasonably in noisy and shorter signals (Chen et al., 2007; Wu et al., 2018; Tian and Song, 2019). As a complexity indicator, a small entropy value is associated with small complexity and great regularity. Studies have shown that the complexity of EMG signals changes in stroke patients with spasticity during voluntary contraction (Dash et al., 2019), and the entropy-based method can reliably and precisely detect the stretch reflex onset (Hu et al., 2018).

Few studies have made an effort on the changes in EMG complexity and spatial distribution of muscle activity when the spasticity is induced. The purpose of this study is to investigate possible alterations in complexity and spatial activation pattern of muscle activity caused by spasticity in comparison with healthy subjects. The combination of HD-EMG amplitude and complexity to study the underlying mechanism of spasticity will provide useful clinical information to give a basis for better therapies and further research on spasticity.

## Materials and Methods

### Participants

Five healthy subjects (three males and two females, 23.6 ± 0.5 years) and three stroke patients participated in this study. The dominant side of all subjects was the right side. The stroke patients with spastic elbow flexor were recruited from The First Affiliated Hospital of Sun Yat-sen University. Table 1 showed the basic information of three stroke patients. The inclusion criteria including: (1) to have hemiplegic elbow flexor spasticity in the range of MAS scores 1–2, (2) to have the ability to perform voluntary elbow flexion, (3) to have sufficient passive ROM of elbow joint, (4) to have intact cognition, auditory and visual sense to understand and follow the experimental instructions, (5) to have no any other orthopedic diseases to impede the neuromuscular function, (6) to have not taken medication for spasticity. This study was approved by the Ethics Committee of The First Affiliated Hospital of Sun Yat-sen University. All subjects signed the informed consent prior to the experiments.

**TABLE 1:** Basic information of stroke patients.

ID	Gender	Age (years)	Time since stroke (months)	Affected side	MAS scores	MMT scores
Patient 1	Male	59	3	Right	1+	3
Patient 2	Male	71	1	Right	1	3
Patient 3	Male	44	3	Right	1	4

*MAS, Modified Ashworth Scale; MMT, Manual Muscle Test.*

### HD-EMG Recording

**FIGURE 1:**
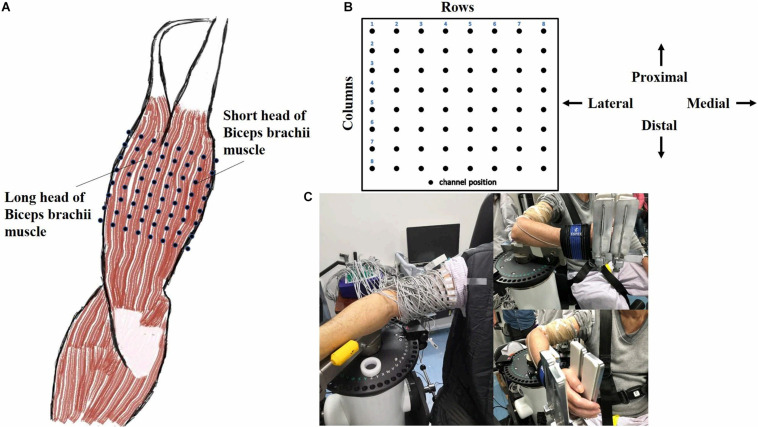
Electrode placement and experimental apparatus. **(A)** A 8 × 8 EMG electrode array was placed over both long and short head of biceps brachii muscle (Right). **(B)** The relative position of the array to the body was presented. **(C)** The experimental apparatus was illustrated in reality operation.

An isokinetic dynamometer system (CSMi, HUMACNORM, United States) was used to perform the passive movement and record the torque at a sampling frequency of 100 Hz. The monopolar HD-EMG signals of both heads of biceps brachii (BB) muscle of the dominant side of healthy subjects or the affected side of stroke patients were acquired simultaneously by the REFA system (TMSi, REFA, Netherlands) at a sampling frequency of 2000 Hz. The 8 × 8 electrode array consisted of 64 separate channels with an electrode’s diameter of 2 mm and an interelectrode distance of 12 mm in both directions, as shown in Figure 1A. Following the columns of the array along with the muscle fiber, the center of the electrode array was placed on the line between the medial acromion and the fossa cubit at one-third from the fossa cubit, and a reference electrode was placed on the styloid process of ulna according to the recommendation of SENIAM. Figure 1B showed the relative position of the electrode array to the body. In order to ensure the signal quality, the conducting gel was applied on the surface of each electrode, and the electrode array was fixed with the aid of an elastic bandage. Before the placement of the electrode array, the skin was abraded and cleaned with abrasive cream and alcohol to reduce noise. For eliminating the influence of gravity, a custom-built fixture holder was used to support the forearm on the anti-gravity position with a foam pad at the bottom of the elbow, as shown in Figure 1C. The forearm was fastened to the dynamometer rig with an elastic belt, which was approximately 3 cm above the wrist. The rotation axis of the dynamometer aligned with the movement axis of the elbow joint.

### Experimental Procedure

The subject sat comfortably on the chair of the isokinetic dynamometer system with the dominant forearm or affected forearm fixed on the custom-built equipment. The initial position of the subject was with the shoulder abduction and elbow flexion in 90°, and the forearm, wrist and fingers were kept in a neutral position. Two straps were crossed in front of the chest and one strap was tied at the waist to stabilize the trunk. Then the subject was familiarized with the whole experimental process before the formal test, including passive movement, and maximal isometric voluntary contraction (MIVC).

In the first stage, the range of elbow passive movement from the starting position was first measured manually. The range of all subjects was 90°, except the patient 1 was 80° because of discomfort, but the angle was not crucial to results. The subject was asked to relax as much as possible, then his/her forearm was passively moved along with the custom-built equipment within the predefined range at four constant angular velocities with this order: 10°, 60°, 120°, and 180°/s. If the velocity is below a certain threshold, the stretch reflex response would not be observed (Wang et al., 2019), so the slow baseline velocity of 10°/s can represent a quiet state of no reflex response. One measuring trial consisted of moving back and forth twice between the predefined range. The process from the starting position to the end position of passive movement was referred to as the passive stretch of BB. When one trial was finished, the subject was relaxed with a 10 s interval time. Three trials were performed at each velocity with a 60 s break before velocity changing. After all velocities were completed, the subject was allowed to take a 3 min rest, then moving on to the next stage.

In the second stage, the MIVC of elbow flexion was performed at an elbow joint angle of 90° flexion. The subject was asked to push the machine with the maximal force for 4 s and was encouraged verbally to ensure maximal contraction. Three trials were performed with a 1 min break between each trial.

### Data Analysis

For the HD-EMG signals recorded in the experiment, a 6th-order Butterworth band-pass filter from 20 to 500 Hz and a 40th-order 50 Hz notch filter were applied to remove the interference noise and power-line interference. After that, the HD-EMG recordings of the first passive stretch of BB were cut out separately according to each stretch velocity task. Then the trial that had the maximum number of effective channels for each velocity was chosen to calculate the feature values:

(1) Root mean square (RMS), the RMS value is used to quantify the intensity of muscle activity. The 2D RMS activation map *AM* with a dimension of 8 × 8 was calculated to visualize the spatial distribution of muscle activity as follows:

(1) $${\mathrm{AM}}_{\mathrm{ij}}=\frac{\frac{1}{M}\,\sum_{m=1}^{M}{\mathrm{RMS}}_{m}\,\left(x_{\mathrm{ij}}\right)}{\mathrm{MIVC}}$$

where $R\,M\,S_{m}\,\left(x_{i\,j}\right)=\sqrt{\frac{1}{N}\,\sum_{n=1}^{N}x_{i\,j}^{2}\,\left(n\right)}$ as used to calculate the RMS value of the *m*-th segment, HD-EMG recordings were divided into *M* segments with a non-overlapping window length of 200 ms in each task, *N* corresponded to the total number of samples in a window length, *x*_*ij*_ as the EMG signal from the channel located at row *i* and column *j* of the electrode array, here channel (1,1) corresponding to the upper left corner of Figure 1B. The maximum RMS value of all non-overlapped windows in three trials was considered as the MIVC amplitude. The amplitude of each pixel in the map represented the normalized activation intensity of the corresponding channel of the electrode array. The yellow color (bright) referred to the maximum activation of the channel while the blue color (dark) referred to the minimum activation of the channel.

(2) Fuzzy entropy (FuzzyEn), the FuzzyEn values of 64-channel were computed as follows (Chen et al., 2009):

Given an *N* samples time series {*u*(1), *u*(2), …, *u*(*N*)}, define *m* to reconstruct the vector:

(2) $$X_{i}^{m}=\left\{u\,\left(i\right),u\,\left(i+1\right),\ldots ,u\,\left(i+m-1\right)\right\}-\bar{u}\,\left(i\right)$$

where $\bar{u}\,\left(i\right)=\frac{1}{m}\,\sum_{k=0}^{m-1}u\,\left(i+k\right)$, *i* = 1, 2, …, *N*-*m*++1.

For a certain $X_{i}^{m}$the $d_{i\,j}^{m}$ is the maximum absolute difference of $X_{i}^{m}$ and $X_{j}^{m}$*j* = 1, 2, …, *N*-*m*+1; *j* ≠ *i*.

(3) $$d_{\mathrm{ij}}^{m}=d\,\left[X_{i}^{m},X_{j}^{m}\right]=\max_{p\in \left(0,m-1\right)}\left|\left(u\,\left(i+p\right)-\bar{u}\,\left(i\right)\right)-\left(u\,\left(j+p\right)-\bar{u}\,\left(j\right)\right)\right|$$

For the given *n* and *r*, the similarity degree $S_{i\,j}^{m}$ of $X_{i}^{m}$ and $X_{j}^{m}$is calculated:

(4) $$S_{\mathrm{ij}}^{m}=\exp\left(-{\left(\frac{d_{\mathrm{ij}}^{m}}{r}\right)}^{n}\right),n=2$$

Define the function C${}_{i}^{m}$ as:

(5) $$C_{i}^{m}\,\left(r\right)=\frac{1}{N-m+1}\,\sum_{j=1,j\neq i}^{N-m+1}S_{\mathrm{ij}}^{m}$$

Then get the Φ^*m*^(r):

(6) $$\Phi^{m}\,\left(r\right)=\frac{1}{N-m+1}\,\sum_{i=1}^{N-m+1}l\,n\,\left(C_{i}^{m}\,\left(r\right)\right)$$

Finally, we can define the FuzzyEn function as:

(7) $$F\,u\,z\,z\,y\,E\,n\,\left(m,r\right)=\lim_{N\to \infty }\left[\ln\,\Phi^{m}\,\left(r\right)-\ln\,\Phi^{m+1}\,\left(r\right)\right]$$

That, for finite datasets, can be estimated by the statistic:

$$F\,u\,z\,z\,y\,E\,n\,\left(m,r,N\right)=\ln\Phi^{m}\,\left(r\right)-\ln\Phi^{m+1}\,\left(r\right)\,\left(8\right)$$

In this study, *m* = 2, *r* = 0.2 ^∗^ STD of the HD-EMG signals.

The same as above, the 2-D FuzzyEn maps would be acquired to visualize the topographic distribution of the complexity of EMG signals for each velocity. What’s more, the average value of RMS and FuzzyEn over 64-channel was calculated, respectively. The data of five healthy subjects were averaged as the healthy group to compare with stroke patients. A linear correlation between the average value of RMS and FuzzyEn of the healthy group and three stroke patients was analyzed, respectively.

## Results

**FIGURE 2:**
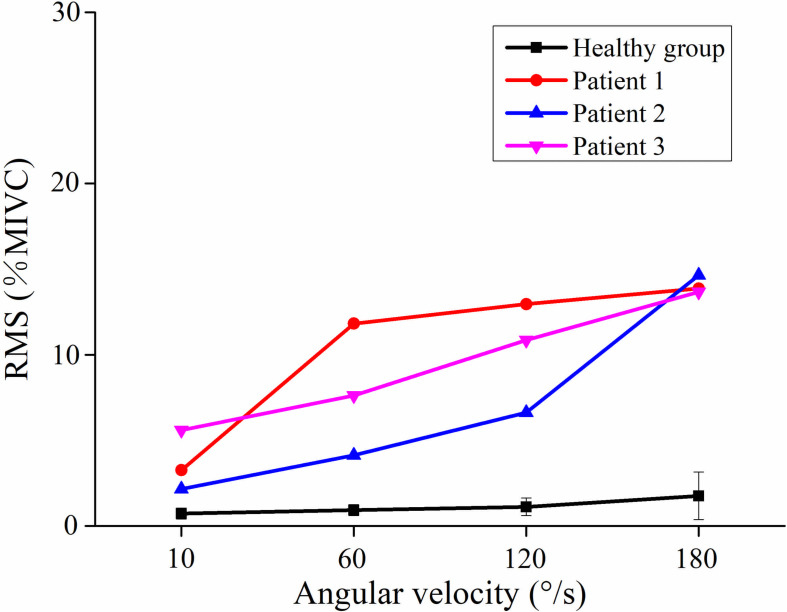
The relationship between the angular velocity of passive stretch and the average value of root mean square (RMS) of the healthy group (healthy group: the average of five healthy subjects) and each stroke patient.

**FIGURE 3:**
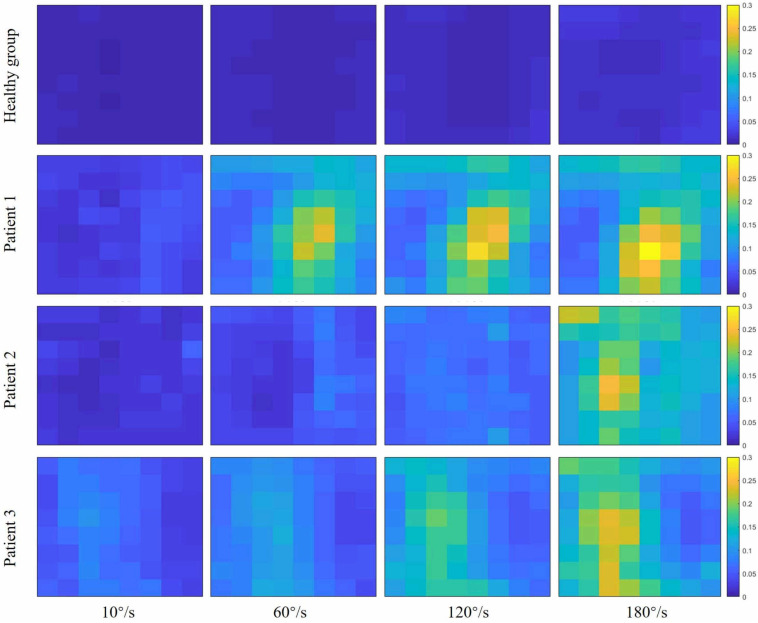
Root mean square (RMS) maps of passive stretch at four angular velocities of the healthy group (healthy group: the average of five healthy subjects) and each stroke patient.

Figure 2 showed the curve of the average value of RMS over 64-channel with the velocity of the healthy group and three stroke patients. The trends of RMS average value in different velocity were different between the healthy group and stroke patients. It was obvious that the RMS value of stroke patients increased linearly with the velocity. The RMS average value of quick stretch (60°/s, 120°/s, and 180°/s) were much greater than that of slow baseline (10°/s) of stroke patients. However, the RMS value of the healthy group did not increase linearly but gradually trends gently. Furthermore, Figure 3 illustrated that the RMS activation maps of the healthy group and three stroke patients at four velocities. The position of the RMS map relative to the body is the same as the position of the electrode array relative to the body. As the increase of velocity, the RMS maps of the healthy group were always kept the dark state. Different from the healthy group, the activation region of stroke patients was expanded with the increasing velocity and the activation was heterogeneous within the muscle. It was noteworthy that the activation areas of the three stroke patients were different.

**FIGURE 4:**
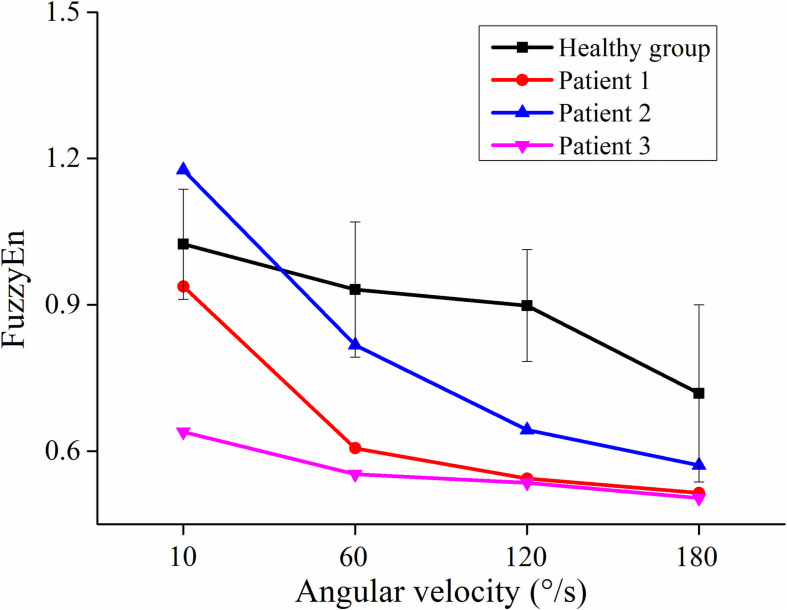
The relationship between the angular velocity of passive stretch and the average value of fuzzy entropy (FuzzyEn) of the healthy group (healthy group: the average of five healthy subjects) and each stroke patient.

**FIGURE 5:**
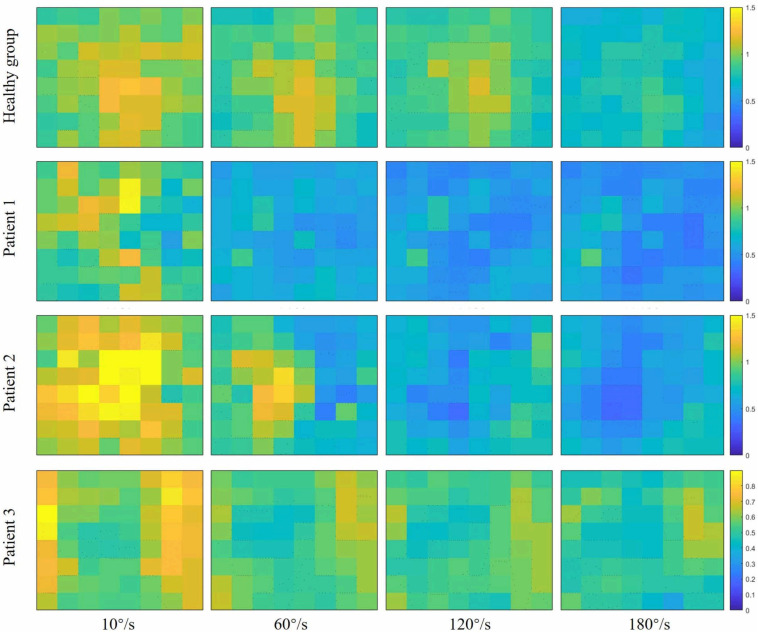
Fuzzy entropy (FuzzyEn) maps of passive stretch at four different angular velocities of the healthy group (healthy group: the average of five healthy subjects) and each stroke patient.

Figure 4 showed the curve of the average value of FuzzyEn over 64-channel with the velocity of the healthy group and three stroke patients. There was a decreasing trend in FuzzyEn value with increasing velocity of the healthy group as well as three stroke patients. But the extent of reduction of stroke patients was greater than that of the healthy group, especially from the slow baseline of 10°/s to quick stretch of 60°/s. Figure 5 illustrated that the FuzzyEn maps of the healthy group and three stroke patients at four velocities, with the same relative position to the body as above. It was clear that the complexity of stroke patients decreased sharply in a large area as the velocity progressed. On the contrary, the complexity of the healthy group decreased slowly in a smaller area.

**FIGURE 6:**
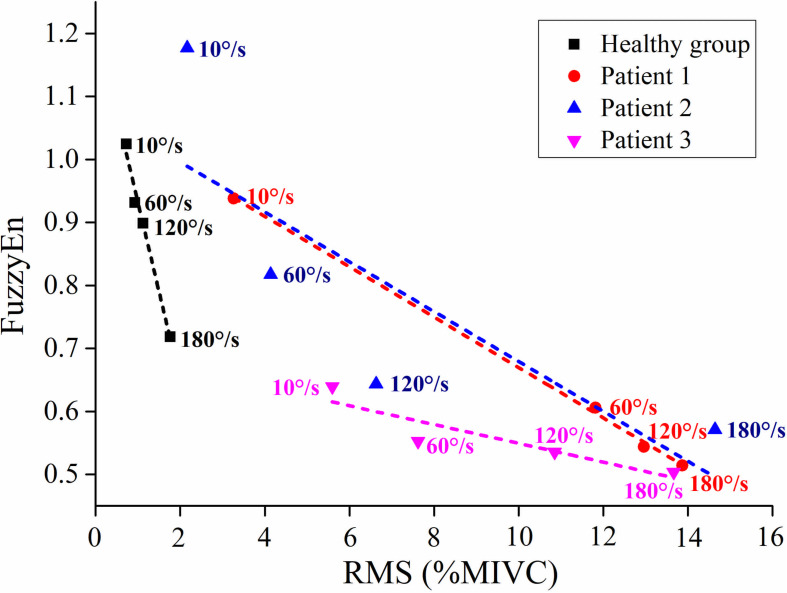
The relationship between the average value of root mean square (RMS) and fuzzy entropy (FuzzyEn) of the healthy group (healthy group: the average of five healthy subjects) and each stroke patient.

As shown in Figure 6, although the RMS value was negatively correlated with the FuzzyEn value in the healthy group and three stroke patients, their rate of change was different. When the quick stretch was carried out, the changes of the stroke patients were greater both for the RMS and FuzzyEn value compared with the healthy group, especially from the 10°/s to 60°/s. In addition, although three patients showed similar trend lines, there were some differences among the three of them in response to the increased stretch velocity. The most obvious reduction in FuzzyEn values was from the slow baseline of 10°/s to a quick stretch of 60°/s for all of them. But in terms of the most obvious increase in RMS values was from 10°/s to 60°/s for patient 1, 120°/s to 180°/s for patient 2, and 60°/s to 120°/s for patient 3.

## Discussion

In order to study the dysfunctional muscle activity resulted from spasticity, case studies of three stroke patients were reported by comparison with healthy subjects. Benefited from the HD-EMG technique, the spatial heterogeneous activation was found within the spastic muscle due to spasticity. Besides, by using FuzzyEn index, it was found that the EMG complexity decreased in response to incremental stretch velocity. The changes in amplitude and complexity may be associated with the high degree of synchronization caused by high MUs recruitment with the emergency of spasticity. The muscle activity was heterogeneous and the activation area was gradually expanded as the velocity progressed, which indicated that the spatial recruitment pattern of MUs was non-uniform in the spastic muscle and this MUs recruitment increased with the reflex response.

### The EMG Amplitude and Spatial Distribution

Compared with the healthy group, the reflex EMG amplitude of stroke patients exhibited a linear increase during the quick passive stretch. The increased reflex response of stroke patients indicated that their excitability of stretch reflex was enhanced pathologically. The previous study showed a velocity of 35°/s is fast enough to induce significant EMG activity in spastic elbow flexor (Lee et al., 2002). The EMG activity of stroke patients increased with the stretch velocity, which was consistent with the definition of spasticity, which was muscle overactivity caused by the velocity-dependent exaggeration of the stretch reflex (Ward, 2012). The stroke patients with spasticity failed to regulate the stretch reflex due to the alterations in the sensitivity of muscle spindle and supraspinal command (Gracies, 2005; Sheean and McGuire, 2009). Therefore, the excessive reflex response to quick stretch resulted in the involuntary activity of spastic muscle. Similar results were reported in previous researches using conventional bipolar EMG, that is, the stretch reflex response of stroke patients was positively correlated with stretch velocity (Kim et al., 2005; Sorinola et al., 2009). However, there have been few articles investigating the spatial distribution of muscle activity during passive stretch. In this study, the spatial inhomogeneous activation and expanding activation area were found in stroke patients by the HD-EMG technique. Distinct areas were activated differentially during passive stretch revealed that the MUs recruitment resulted from spasticity is non-uniform within the spastic muscle. The spatial distribution of muscle activity is related to the distribution and recruitment strategy of MUs within a muscle (Holtermann et al., 2005; Rojas-Martínez et al., 2013; Abboud et al., 2018). The high-threshold MUs degenerated after stoke so that the MUs recruitment caused by the stretch reflex was mainly to recruit the smaller low-threshold MUs (Kallenberg and Hermens, 2011). Thus, the modulation of MUs recruitment could be associated with heterogeneities in muscle activation. Meanwhile, the gradually broadening activation area indicated that the number of recruited MUs would increase with the increase of velocity. In the three stroke patients, the activation area was different indicated that the spatial activation pattern of a spastic muscle might vary between individuals. A previous study showed that the MUs recruitment pattern resulted from the stretch reflex changed depending on the level of spasticity (Hu et al., 2018).

### The Complexity of HD-EMG Recordings

In the process of increasing stretch velocity, the EMG complexity of stroke patients decreased more than that of the healthy group. The induced stretch reflex is accompanied by the recruitment of MUs (Kallenberg and Hermens, 2011; Kosar et al., 2012). It has been confirmed that the MUs were activated by the same reflex mechanism that would result in the synchronism of EMG signals (Evans et al., 1983). Therefore, the decrease in complexity may be the result of the increased synchronization of EMG due to stretch reflex. In addition, researches had shown that the motoneurons were synchronized when they were recruited by the stretch reflex (Schuurmans et al., 2009; Finley et al., 2013), which would also lead to the synchronous activation of MUs. Studies have shown that the balance between the inhibitory and excitatory tract that controls the stretch reflex is disrupted after a UMN lesion resulting in a disinhibition of stretch reflex, and only the origin of the inhibitory track is under the control of the cortex (Ward, 2012; Trompetto et al., 2014). In consequence, the exaggerated reflex response of stroke patients would recruit more MUs leading to higher synchronization. The faster the stretch velocity, the more synchronization, so the complexity is sensitive to the stretch velocity. On the other hand, it could be speculated that the reduction in the complexity of stroke patients was also related to the decreased information from the cerebral cortex to the spinal cord. Due to pathological reasons, there is less information from the cerebral cortex to the spinal cord, so the information from the spinal cord to the muscles is also reduced. Some researchers reported that the neural drive to muscles was positively correlated with the complexity of EMG (Kamavuako et al., 2012; Dash et al., 2019). It was interesting to note that the complexity of patient 1 and patient 2 decreased rapidly when the stretch velocity changed from the baseline of 10°/s to a quick stretch of 60°/s, and much slower from 120°/s to 180°/s, while the opposite phenomenon occurred in the healthy group. Different from the healthy group, stroke patients were sensitive to the stretch velocity. Once the quick stretch was performed, the stretch reflex was induced to cause a large amount of EMG synchronization, so the complexity from the baseline to quick stretch decreased rapidly. However, the change of patient 3 from baseline to quick stretch was slower than them. It might be due to the lower level of spasticity, causing lower sensitivity to quick stretch. For different quick stretch velocities, the difference was only in the degree of induced spasticity, so the much slower decrease from 120°/s to 180°/s may be due to the limited number and firing rate of recruited MUs. On the contrary, the change from 120°/s to 180°/s was most obvious for the healthy group, which may be because the velocity was too fast and exceeded the threshold at which healthy subjects can control their stretch reflex. When the BB was stretched faster, the complexity of stroke patients decreased rapidly in a clearly large area. The brightest areas of RMS maps corresponded to the darkest areas of FuzzyEn maps, so it might be hypothesized that the spatial heterogeneity of complexity is associated with the spatial distribution of muscle activation.

In comparison with the healthy group, stroke patients showed evident differences in RMS and FuzzyEn when the fast velocity was used. The negative correlation between them implied that the higher amplitude was associated with lower complexity. Moreover, the different responses of the three stroke patients to the increased stretch velocity suggested that patients may have different sensitivity to stretch velocity due to the different levels of the hyperexcitability of the stretch reflex. To our knowledge, this is a prospective study showing that the exaggeration of the stretch reflex is accompanied by an increase in the recruitment and synchronization of MUs. The application of the HD-EMG technique can provide rich spatial information to facilitate understanding the physiological mechanism underlying spasticity on the level of MUs.

### Limitations

Three cases might not be sufficient for all stroke patients with different severity of spasticity, and the ages of stroke patients and healthy subjects were not exactly matched. Further research is needed to increase the data of stroke patients with various levels of spasticity and the date of age-matched healthy subjects to obtain more comprehensive information and to further better investigate the physiological mechanisms underlying the spasticity. Meanwhile, the normalization of RMS is related to the results of EMG amplitude. In addition, the HD-EMG decomposition technique would help to provide direct evidence regarding the underlying mechanism of spasticity on the MUs level (Holobar and Zazula, 2007; Chen and Zhou, 2016). From this point of view, the findings in our current work can provide guidance for HD-EMG decomposition, which will be completed in the future.

## Conclusion

The neuromuscular response to passive stretch of stroke patients with spasticity is different from that of healthy subjects, which is reflected in the increased amplitude and decreased complexity of EMG activity. Furthermore, the involuntary muscle activity due to spasticity is not only manifested in the EMG amplitude, but also in spatial distribution. It would be beneficial to further pathological research and clinical management of spasticity.

## Data Availability Statement

The raw data supporting the conclusions of this article will be made available by the authors, without undue reservation.

## Ethics Statement

The studies involving human participants were reviewed and approved by the Ethics Committee of The First Affiliated Hospital of Sun Yat-sen University. The patients/participants provided their written informed consent to participate in this study. Written informed consent was obtained from the individual(s) for the publication of any potentially identifiable images or data included in this article.

## Author Contributions

TX, YL, YZ, and ZL collected the data. TX, CJ, and NT analyzed the data and drafted the manuscript. HY and RS revised and determined the final manuscript.

## Conflict of Interest

The authors declare that the research was conducted in the absence of any commercial or financial relationships that could be construed as a potential conflict of interest.
